# A methodological framework for building interoperable sorption databases: application to cesium sorption in crystalline rocks

**DOI:** 10.12688/openreseurope.23714.1

**Published:** 2026-05-26

**Authors:** Tiziana Missana, Miguel García-Gutiérrez

**Affiliations:** 1 Physical Chemistry of Actinides and Fission Products Unit, Centro de Investigaciones Energeticas Medioambientales y Tecnologicas, Madrid, Community of Madrid, 28040, Spain

**Keywords:** Experimental databases, Knowledge Management, Sorption Modelling, Safety Assessment, Radioactive Waste

## Abstract

Databases are key to knowledge management and to implement FAIR (Findable, Accessible, Interoperable, and Reusable) principles in scientific research. However, most experimental sorption data remain underused owing to heterogeneous reporting practices, incomplete metadata, or lack of standardization. Furthermore, existing sorption databases often focus primarily on distribution coefficients (K _d_), with a limited description of the broader physicochemical context, thereby restricting their applicability to comprehensive geochemical analyses.

This study proposes a structured framework for the generation and organization of sorption data to guide experimentalists in producing reusable datasets. The framework defines key principles for the construction of sorption databases, including the types of data to be collected (e.g., distribution coefficients, K _d_, and relevant geochemical parameters), as well as the distinction between measured and derived data.

The methodology is demonstrated using experimental sorption data on cesium retention in crystalline rocks, comprising approximately 1000 K _d_ values, complemented by detailed physicochemical information on the experimental conditions, aqueous chemistry, and solid properties.

Example applications, ranging from statistical analyses to sorption modelling, illustrate the potential of the approach and highlight the added value of comprehensive and well-structured datasets. The proposed framework can be further developed to foster more systematic and collaborative approaches to experimental data management, and can be applied to other radionuclides and materials.

## 1. Introduction

Experimental data on radionuclide (RN) sorption are essential for geochemical modelling and safety assessment of radioactive waste repositories. However, despite decades of experimental work, there is still no consensus on the best practices for data collection and reporting aimed at facilitating interoperability or their reuse in modelling workflows.

Several international initiatives have sought to compile RN sorption data, mainly focusing on distribution coefficient, K _d_, values. The NEA sorption database developed by the OECD-NEA ( Ruegger and Ticknor, 1992) provided a basic source of information describing sorption of key nuclides for a large variety of solid and liquid phases. The Japan Atomic Energy Agency (JAEA) developed a sorption database (JAEA-SDB) focusing on data quality for performance assessment ( Sugiura et al., 2020). The Korea Atomic Energy Research Institute (KAERI) developed a sorption database (SDB-21C) that includes a large amount of sorption data, offering functions for adding, searching, and graphically representing data ( Jung et al., 2001; Lee et al., 2011). The Belgian agency for radioactive waste, ONDRAF/NIRAS, leads the compilation of a sorption database, particularly focusing on clay and cementitious materials, with the primary goal of supporting their safety assessments. The Paul Scherrer Institute (Switzerland) generated a database grounded on the analysis of data using geochemical and mechanistic modelling, focused in the Opalinus clay ( Manrich et al., 2025).

Other types of databases, such as RES ^3^T (Rossendorf Expert System for Surface and Sorption Thermodynamics) ( Brendler et al., 2003), offer thermodynamic sorption data primarily for mechanistic modelling purposes. Lawrence Livermore National Laboratory (LLNL) developed the LLNL Surface Complexation/Ion Exchange (L-SCIE) database, which also includes a data-fitting workflow to efficiently optimize surface complexation reaction constants using multiple surface complexation models ( Zavarin et al., 2022).

Each database has internal coherence and a different degree of completeness, depending on its specific focus. Nonetheless, the compilation of previously published K _d_ data alone is not sufficient; structured approaches to experimental data curation are required from the earliest stages of experimental design to ensure traceability, consistency, and reusability. In an ideal context, the concept of a usable database should be considered from the very beginning of any experimental activity, and guidance on how to collect, manage, and transfer the data is essential.

In this work, a structured methodological framework for the construction of experimental sorption databases and an internal structure that facilitates a direct link to the main physical and chemical characteristics of the system are proposed. For each sample, not only is the K _d_ value recorded, but also, when possible, the characteristics of the experiment (radionuclide concentration, contact time, solid-to-liquid ratio, rock grain size), the chemistry of the water (pH, concentration of main ions, electrical conductivity), and mineralogy of the rock. Additional entries can be included for basic calculations, such as the percentage of adsorption, final radionuclide concentration, sorption site density, and other estimations. The resulting dataset supports a range of applications, from statistical analyses to sorption modelling.

This analysis focuses on cesium sorption data in crystalline rocks, as Cs has relatively simple chemistry and well-characterized sorption behaviour. The first version of the experimental dataset was deposited in ZENODO ( Missana and García-Gutiérrez (2025), https://zenodo.org/records/17930595). Indeed, the framework proposed here is not intended as a final solution, but as a flexible and evolving approach that can be progressively improved through community contributions and adapted to the different systems under study.

## 2. Cesium sorption in crystalline rocks

The isotope ^137^Cs (half-life, ~30 years) is an important fission product of the irradiation of uranium-based fuels, and its inventory in nuclear waste is high. It is a radionuclide of special environmental interest, as it has been released to soils and waters upon past nuclear accidents or weapon testing ( Steinhouser et al., 2014; Yaqub et al., 2024). Several studies have been conducted on Cs sorption, especially for clayey materials, but relevant literature also exists on sorption in crystalline rocks ( Missana and García-Gutiérrez, 2013; Muuri et al., 2016, 2017; Tsai et al., 2009). Cs was selected because it has simple chemistry and its sorption behavior is well documented.

Crystalline rocks (CR) are suitable for radioactive waste storage, and the first repositories in Europe, Finland, and Sweden were built in CR. They consist of various minerals, mainly quartz, feldspars, and micas, along with other accessory minerals. The CR is sparingly fractured, and fractures can be partially filled with secondary minerals formed through weathering and alteration of the primary rock. The composition of these fillings largely depends on the original rock type, and can be composed of quartz, secondary micas, iron oxyhydroxides, oxides, calcite, carbonates, or clay minerals.

As crystalline rocks are characterized by mineralogical heterogeneity, retention processes may comprise ionic exchange (with constant-charge minerals such as micas and clay minerals) and/or surface complexation (with minerals exhibiting pH-dependent surface charge, e.g., quartz, feldspar, or any mineral showing amphoteric sites). Cesium interacts with natural solids through both cation exchange ( Sawhney, 1970) and specific adsorption, which depend on the properties of the solids, that is, their cation exchange capacity (CEC), and the presence of mica-like minerals, which present sorption sites with high selectivity for alkali cations ( Sawhney, 1970, 1972; Poinssot et al., 1999; Zachara et al., 2002).

In crystalline rocks, mica minerals exhibit a low cation exchange capacity (CEC) owing to the presence of non-exchangeable cations. However, weathering promotes sorption via frayed-edge sites (FES), which are highly selective for Cs and other alkali ions. In illite-rich or weathered micas, Cs retention is dominated by FES, which prevails at low concentrations, whereas ion exchange becomes significant at higher concentrations ( Missana et al., 2014).

Biotite and other mica group minerals can exhibit high affinity for cesium (especially when it is present at a low concentration) owing to their FES, and quartz and feldspars show, in principle, lower sorption capacities.

Crystalline rocks were selected because their diverse mineralogy and inherent heterogeneity provide a wide range of chemical environments that are useful for data analysis.

## 3. Methods

### 3.1 Sorption data compilation

The initial sorption database was compiled from extensive experimental work conducted at CIEMAT over the last few decades. This includes the raw experimental data obtained from different projects. The database systematically integrates the distribution coefficient (K _d_) values derived from batch sorption experiments on granitic rocks from different European sites and their constituent minerals (quartz, biotite, muscovite, and feldspar).

***3.1.1 Contents of the current database***

The first version of the database (Version v1.0) is available in ZENODO, with the following DOI: 10.5281/zenodo.17930594. It is in Excel file format (Cs_sorption_in CrystallineRock_V1_Dec25.xlsx) and is composed of three sheets:

1. Readme
2. Cs sorption data on minerals (Minerals)
3. Cs sorption data on granitic rock (Granite).

The sheet *Minerals* the sheet *Granite* contained 436 and 577 data lines (experimental entries), respectively.

The main parameter under analysis was the distribution coefficient K _d_, which represents the capability of a solid to retain a contaminant. It is defined as the ratio between the concentration of sorbate in the solid and the concentration of the contaminant that remains in the solution when sorption equilibrium is reached:

$$K_{d}=\frac{C_{\text{in}}-C_{\mathrm{fin}}}{C_{\mathrm{fin}}}\cdot \frac{V}{m}$$

C _in_ is the initial contaminant concentration and C _fin_ is the quantity of contaminant remaining in the liquid. V is the volume of water and m is the mass of the solid. The quantity C _in_-C _fin_ represents the contaminant adsorbed on the solid.

K _d_ values were expressed in mL·g ^−1^ and determined using the batch sorption technique with crushed rock. All tests were performed at room temperature. K _d_ values were obtained from different types of experiments: single-point K _d_, sorption kinetic tests (as a function of time), sorption isotherms (as a function of radionuclide concentration), and sorption edges (as a function of pH).

It must be noted that the term K _d_ is valid only if the sorption equilibrium is reached. If it is not, it should be called the distribution ratio, R _d_. In this study (and in the database), we will always refer to the measured ratio as K _d_.

The starting hypothesis of this work is that for an appropriate analysis of the experimental K _d_ values, they must be correlated with the key physicochemical properties of the solid phase and equilibrium water chemistry. This approach seeks a systematic categorization of data by means of the creation of a structured “matrix” according to main characteristics of the solids and solutes and accounting for the experimental conditions in detail.

The first column of the database file contains general information about the samples with

- **Mineral type:** Referring to the type of mineral used (biotite, quartz), the suffix -n; −s was added to distinguish whether the tests were performed in saline (−s) or non-saline (−n) solutions. The considered limit is 0.01 M (n < 0.01 M; s ≥ 0.01 M).
- **Site & type of granite:** The rock sample was defined according to the site (Grimsel, Äspö, etc.) and other details available on the type. The suffix -n; −s was added to distinguish whether the tests were performed in saline (−s) or non-saline (−n) solutions.

The second block of information contains the experimental conditions: contact time, solid-to-liquid ratio, initial sorbate concentration, and sample grain size. It could be extended to other required information, such as the type of separation (filtration/centrifugation), and includes more details of interest. In the present study, all samples were separated by centrifugation at g > 20000.

The block of columns related to the experimental conditions includes:

- **Atmosphere (Atm)**: Indicates whether the tests were performed under oxic (OX) or anoxic (AN) conditions.
- **Time (t):** Contact time in sorption experiments it is expressed in days.
- **Initial Cs concentration** (Cs _INI_): Expressed in mol·L ^−1^.
- **Log (Cs**_**INI**_ **):** It sometimes facilitates data plotting.
- **g/L:** Solid to liquid ratio expressed in g·L ^−1^.
- **V/m**: Liquid to solid ratio expressed in ml·g ^−1^.

The third block contains the main information on the chemistry of the contact electrolyte.

- **pH**_**final**_ **:** pH measured at the end of the sorption test
- **K:** potassium concentration in mol·L ^−1^
- **Na:** sodium concentration in mol·L ^−1^
- **Ca + Mg** (Div) = concentration of the sum of Ca and Mg in mol·L ^−1^; Div stands for “divalent.”
- **Cl:** chloride concentration in mol·L ^−1^
- **HCO**_**3**_^**−**^: bicarbonate concentration in mol·L ^−1^
- **SO**_**4**_^**2−**^: sulfate concentration in mol·L ^−1^
- **El.Co**: electrical conductivity in μSm·cm ^−1^

In most cases, these data correspond to the initial contact water; however, when data on the equilibrium water upon the experiments were available, they were preferred. It is of interest to know the quantity of the sorbate naturally present (if it is) both in water and in the solid. When available, the natural content of the element that we are considering as sorbate, in this case [Cs], should be included to correct the K _d_ values, if needed.

The fourth block contained the main properties of the solid and its mineralogy. The composition of each sample might not always be experimentally determined; thus, sometimes data must be obtained from the literature, and this should be clearly indicated. The information included in this block is as follows:

- **BET**: N _2_-BET surface area in m ^2^·g ^−1^
- **CEC:** Cation exchange capacity in meq/100 g
- **Q**: Content in wt% of quartz
- **Feldspars (Fd)**: content in wt% feldspars. They can be divided into two types.
  o Potassic feldspars, **FdK**, as *orthoclase and microcline*
  o Others as *plagioclase and albite*
- **Micas (Mi):** content in wt% of micas including biotite, chlorite, muscovite, and possible traces of clays
- **Others:** includes some other minerals as, for example, hornblende.
- **Size:** This represents the size of the mineral used for sorption tests in millimeters. Often, these values correspond to the upper limit of the fraction used (i.e., 0.064 mm is <0.064 mm).

The following block refers to the experimental sorption data:

- **K**_**d**_ **:** distribution coefficient in mL·g ^−1^( **Log (K**_**d**_ **)**, is calculated)
- **FIN [Cs]:** final concentration of cesium in solution in mol·L ^−1^ **(**Log (Cs _fin_) was calculated).
- **Log (Cs**_**ads**_ **/g):** Cs adsorbed por unit mass (mol·g ^−1^) is calculated

In the experimental database, it is possible to include other calculated parameters, such as K _d_ values normalized to the surface area (K _d_/BET) or the site density, which can be estimated from the CEC and N _2_-BET values.

Whenever possible, the reported values should be accompanied by estimates of experimental uncertainty. The errors in the K _d_ values were not added to the first version of the database but were incorporated asap. In future versions, suspicious points will be flagged as *anomalous.* The present database will be updated periodically according to the new data available or suggestions for improvement.

### 3.2 Selected crystalline rocks

***3.2.1 Spain***

The first large-scale *in-situ* study of Spanish crystalline rocks was the *El Berrocal* Project (1988–1995), funded by the European Commission, and was conducted at an abandoned uranium mine within a granitic batholith of the Spanish *Sistema Central.* The project investigated the migration of natural radionuclides (U, Th, and their decay products) through fractured granite using in *in-situ* experiments supported by laboratory analyses ( Rivas et al., 1995, 1997; Gómez et al., 1996) including sorption and migration tests ( García-Gutiérrez, 1994; García-Gutiérrez et al., 1997).

Between 1997 and 2000, ENRESA restored uranium mines in Andalusia and Extremadura while launching R&D programs on rock and deep groundwater characterization ( Buil, 2002; Pérez-Estaún et al., 2002); geochemical processes ( Gómez, 2002), and radionuclide transport modelling ( Marcuello et al., 2006). A key example of this work is the *Los Ratones* mine **,** in the Albalá granitic pluton (Cáceres, Spain), part of the Central Iberian Zone of the Hesperian Massif. CIEMAT conducted many tests for the complete characterization of the granites from the *Los Ratones* mine, including sorption tests. Sorption data and sample details were obtained from García-Gutierrez et al. (2000).

Data from Spanish crystalline rocks in the database include:

o Fresh and altered granite from *El-Berrocal
*
o Fresh and altered granite from *Los-Ratones* site
o Fracture filling materials.

***3.2.2 Switzerland***

Over the past two decades, the CIEMAT has participated in numerous international projects on deep geological repositories in crystalline rocks. To demonstrate the feasibility of Spain’s repository concept—and specifically to develop and validate the engineered barrier system (EBS) in such host rock—ENRESA launched the FEBEX (Full-scale Engineered Barrier Experiment) project ( Huertas et al., 2000) at the NAGRA’s Grimsel Test Site (GTS).

From the construction of the *in-situ* test in the FEBEX tunnel until its dismantling in 2012 ( Lanyon and Gaus, 2016), extensive studies have been conducted on tunnel geology, groundwater chemistry, and the potential effects of bentonite, including salinity changes in rock porewater and clay colloid formation, and at the bentonite/rock interface.

Furthermore, as part of the European FUNMIG (Fundamentals of Radionuclide Migration) project in 2005, two boreholes (FUN1 and FUN2) were drilled parallel to the FEBEX gallery, 30 cm and 60 cm from the bentonite surface, for groundwater sampling and colloid/chemical analyses ( Missana et al., 2009). The sorption properties of nearby materials were also studied. CIEMAT further contributed to other GTS programmes, such as the CRR (Colloid and Radionuclide Retardation) and CFM (Colloid Formation and Migration) projects in the Migration Tunnel, where the sorption behavior of different radionuclides in geological materials was studied ( Missana and Geckeis, 2006).

Therefore, the materials included in the database are the following:

o Central Aare granite
o Grimsel granodiorite
o Mylonite (from the Migration tunnel)
o Lamprophyre (from the FEBEX gallery)

***3.2.3 Sweden***

The Äspö Hard Rock Laboratory (Äspö HRL) was a research facility dedicated to studies supporting the development of Sweden’s final repository for spent nuclear fuel. Located in the Simpevarp area of Oskarshamn Municipality, the laboratory lies on the island of Äspö, approximately 30 km north of Oskarshamn’s center and near local nuclear power plants. The underground section of the facility decreased to a maximum depth of 460 m. The bedrock at the site is dominated by two crystalline rock types: Äspö diorite (quartz monzodiorite to granodiorite, porphyritic) and Äspö granodiorite (granite to quartz monzodiorite, generally porphyritic). Detailed descriptions of these rocks have been provided by Johnsson et al. (1998).

During the EC – CROCK (Crystalline Rock retention processes) project ( Rabung et al., 2014), CIEMAT performed studies in different materials also including Äspo granite ( Missana and García-Gutiérrez, 2013; Missana et al., 2019). The data selected for the databases are on Äspö diorite.

***3.2.4 Germany***

More recently, within the framework of the EURAD-2 (European Partnership on Radioactive Waste Management) in the RAMPEC ( *Radionuclide Migration under Perturbed Condition*) Project, CIEMAT initiated studies on crystalline rocks to investigate the effect of parameters like the grain size or mineral composition on radionuclide retention under dynamic conditions. To this end, it was decided to purchase rocks and minerals of different grain sizes. The selected supplier was the commercial brand *Kremer Pigmente GmbH & Co.*, which provided granite and gneiss from Germany, although the exact region of origin of the materials was not specified. Granite and gneiss samples were obtained for several grain sizes; however, the database currently includes only data related to Kremer grey granite.

***3.2.5 Separated minerals***

Data for the separated minerals (biotite, muscovite, K-feldspar, and quartz) were obtained mainly from minerals extracted from Spanish granites. Sorption studies were conducted within the EC-CROCK Project ( Missana et al., 2019). Commercial materials (e.g., Kremer) were also used.

### 3.3 Aqueous solutions

The composition of the solutions in equilibrium with the crystalline rocks was very different depending on the formation. However, three main types of water were considered in the experiments, representing the main conditions existing in the GTS ( Missana and Geckeis, 2006), Äspö ( Rabung et al., 2014) and Spanish granitic formations ( Gómez et al., 2006).

Table 1 shows the typical composition of natural water from these sites. The typical porewater of the GTS, at the Migration tunnel, has a low salinity and alkaline pH. Groundwaters from Spanish granitic formations have relatively low salinity, although not as low as that of GTS waters. The water from the Äspö site was variably influenced by seawater, resulting in a much higher ionic strength. Grimsel and Äspö waters can be considered possible natural extremes of aqueous solution chemistry. Whenever possible, sorption tests were performed using natural pore water extracted directly from the site. Commercially available granitic water was used when it was not feasible. In some cases, simplified synthetic solutions simulating low- or high-salinity water have also been employed. When exchanged materials (Na or K) were used for the sorption tests, a simple electrolyte was used (NaClO _4_, KCl). When available, the composition of the solution upon equilibrium with solids was determined ( Missana et al., 2019)

**Table 1.** Examples of mean chemical composition of crystalline waters from different sites (GTS, Äspö and Spanish reference granite waters).

Composition	GRIMSEL (Switzerland) ( Missana and Geckeis, 2006)	ÄSPÖ (Sweden) ( Rabung et al. 2014)	LOS RATONES (Spain) ( Gómez et al. 2006)
**Na** ^**+**^ **(mg·L** ^**-1**^ **)**	16	1700	115
**Ca** ^**2+**^ **(mg·L** ^**-1**^ **)**	7	1200	7
**Mg** ^**2+**^ **(mg·L** ^**-1**^ **)**	<1	105	7
**K** ^**+**^ **(mg·L** ^**-1**^ **)**	<1	13	3
**Cl** ^**-**^ **(mg·L** ^**-1**^ **)**	2	4700	26
**SO**_**4**_^**2-**^ **(mg·L** ^**-1**^ **)**	6	400	9
**F** ^**-**^ **(mg·L** ^**-1**^ **)**	6	1.2	0
**HCO**_**3**_^**-**^ **(mg·L** ^**-1**^ **)**	30	12	321
**pH**	9.5±0.2	7.5±0.2	9±0.2
**El. Conductivity (μS·cm** ^**-1**^ **)**	102	13000	578

.

## 4. Results

### 4.1 Cs sorption data in granite

***4.1.1 Data visualization***

Figure 1 shows all the Cs sorption data for granite. As the data span several orders of magnitude in the Cs initial concentration (Cs _in_), the clearer form to plot the entire dataset is the log-log plot of the Cs adsorbed concentration (C _ads_) in the solid *vs.* the aqueous concentration remaining in the liquid at equilibrium (C _fin_).

**Figure 1.**
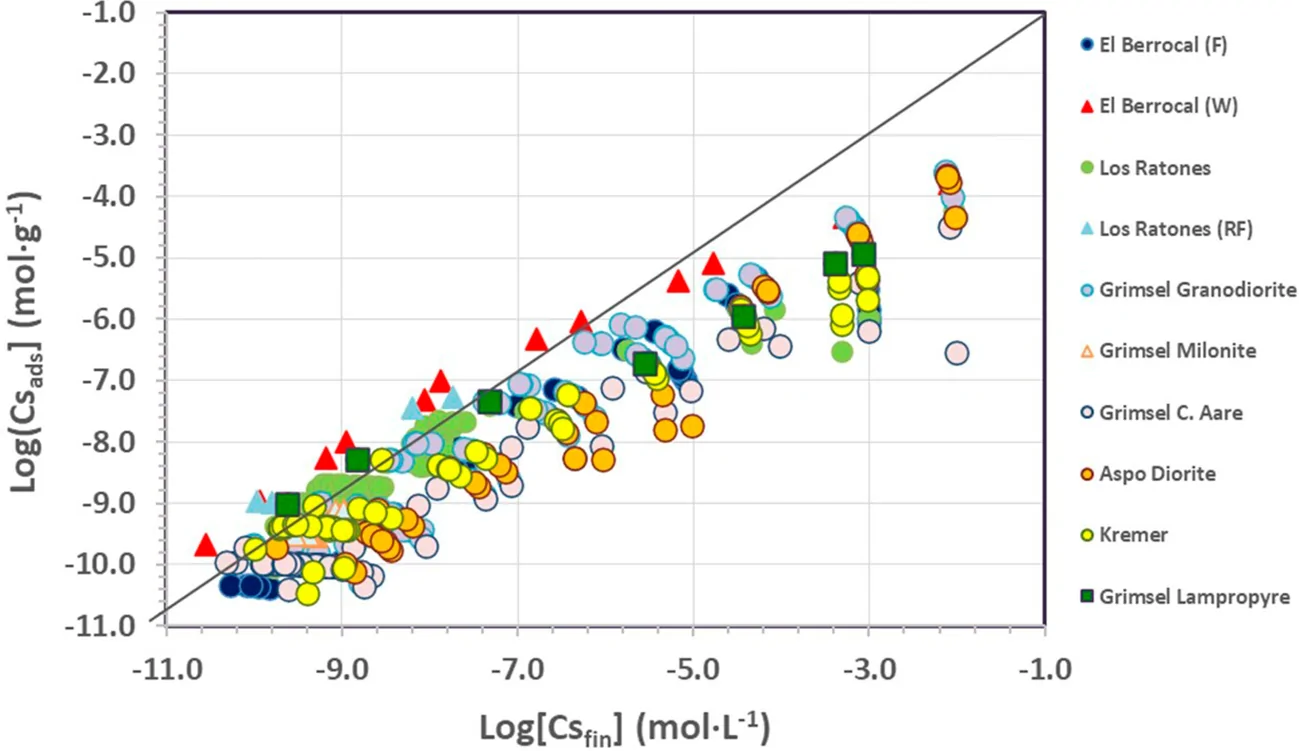
Log-log representation of all Cs sorption data obtained in granite.

The main observation is that for most datasets, Cs sorption is not linear over the range of investigated concentrations, with the slope of the log-log curve less than 1. Non-linear sorption means that K _d_ is not constant but concentration-dependent; in this case, K _d_ decreases as Cs concentration increases, reflecting variable sorption affinities at different sites and/or progressive saturation of sorption sites. This is a fundamental point during the analysis of all the data because the Cs concentration is the parameter that results in higher variability in sorption values and somewhat limits the results of statistical analysis. These observations can guide the successive clustering of experimental data, considering only those within a range of initial Cs concentrations or other characteristics.

Defining subsets may lead to a clearer and more precise description of the results. An example is shown in Figure 2, where the data are plotted as a function of water salinity. Clustering was performed considering saline or non-saline waters (−s,-n), considering the limit of ionic strength of 0.01 M.

**Figure 2.**
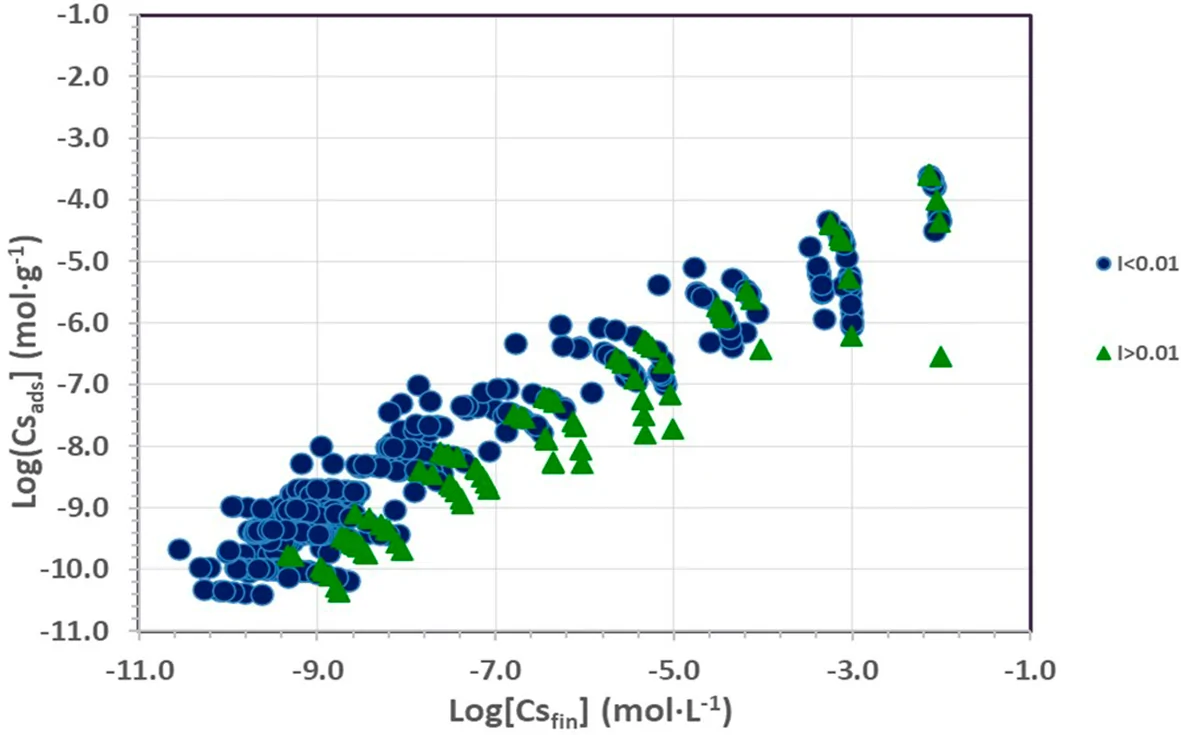
Log-log representation of Cs sorption data in granite differentiating: (●) low saline and (▲) high saline solutions.

Figure 2 shows that the sorption clearly depends on this parameter, especially at low Cs concentrations. In a saline environment, the maximum K _d_ measured was ~350 mL·g ^−1^ but the median was lower than 100 mL·g ^−1^ at all Cs concentrations.

At high Cs concentrations, the influence of salinity on the results became minimal as the distribution coefficient (K _d_) decreased markedly under all salinity conditions (K _d_ ≤ 10 mL·g ^−1^), and the uncertainty also increased. This further emphasizes that Cs concentration is the dominant factor controlling the sorption behavior.

***4.1.2 Basic statistics***

The primary objective of this analysis is not to conduct an exhaustive statistical study but to present a basic study that highlights the potential usability of the collected data. The exploratory data analysis goal is to obtain an initial understanding of data distribution and variability, and above all, potential correlations between parameters. This provides hints for further investigations and organizes the modelling part to refine the interpretation of cesium retention mechanisms.

The initial analysis performed for these examples includes:

- Descriptive statistics (mean, median, standard deviation, variance, min, max) were calculated for the K _d_ values and selected variables (pH, ionic strength, Cs concentration, etc.).
- Boxplots were used to define the characteristic K _d_ variations in each material using the Interquartile Range (IQR) method.
- Correlation matrices (Pearson and/or Spearman) were constructed to evaluate the strength and direction of the relationships between the variables, considering the three main blocks (experimental, water properties, and solid properties).

According to the initial analysis, the clustering of data exhibiting similar sorption behavior may be required to facilitate the interpretation of mineralogical or site-specific effects.

*4.1.2.1 Descriptive statistics*

A summary of the mean, median, standard deviation, minimum, and maximum values for the main parameters analyzed for Cs sorption in granite is shown in Table 2, which shows the wideness of the explored experimental conditions, including the large differences in the chemistry of the systems in terms of both water and solid properties.

**Table 2.** Descriptive statistic of the entire set of data for granite.

Index	time (days)	[Cs]	pH	[K] (mg/L)	[Na] (mg/L)	[Ca + Mg] (mg/L)	BET (m ^2^/g)	CEC (meq/100 g)	Q (%)	FdK (%)	Mi (%)	Size (mm)	g/L	K _d_ (mL/g)
**Mean**	19.68	3.73E-04	7.21	98.43	315.20	160.45	2.18	1.78	31.70	21.53	17.08	0.93	26.78	736.60
**Std**	25.65	1.72E-03	0.81	574.88	694.75	362.83	5.85	3.26	11.67	8.56	8.89	1.10	42.93	1186.38
**Min**	0.00	1.00E-09	3.52	0.00	1.50	0.00	0.01	0.10	14.00	0.80	6.30	0.06	1.05	0.03
**0.25**	10.00	5.47E-09	6.74	0.00	8.30	6.78	0.09	0.66	27.00	15.00	12.00	0.10	10.00	69.89
**0.5**	14.00	5.37E-08	7.10	1.10	13.00	7.00	0.89	0.92	31.00	18.50	14.10	0.50	10.06	313.53
**0.75**	14.00	9.91E-07	7.72	2.10	15.00	57.00	2.78	1.20	40.80	28.00	18.00	1.00	20.00	939.86
**Max**	240.00	9.85E-03	9.90	3900.00	2299.00	1162.00	40.20	19.42	67.50	41.30	46.50	4.00	250.00	9931.54

The experimental entries included tests with a duration from 0 to 240 days, with an initial Cs concentration ranging from approximately 1·10 ^−9^ to 1·10 ^−2^ M. The grain size of the crushed rocks varied from 0.063 to 4 mm, and the solid-to-liquid ratio varied from 1 to 250 g/L. Not all the parameters were included in the example. Therefore, the variation in K _d_ values is expected to be large and spans from approximately 0 to 1·10 ^4^ mL/g.

*4.1.2.2 Box-plots*

To better understand the existing variability and detect possible outliers, boxplot graphs ( Figure 3) were used. Each boxplot summarizes the spread of K _d_ values for a given material, which contains half of the data, belonging to the interquartile range (IQR) from the 25 ^th^ (quartile 1, Q1) to 75 ^th^ (Q3). The horizontal line inside the box indicates the median K _d_ (Q2). The whiskers extend from Q1–(1.5·IQR) and Q3 + (1.5·IQ3), considering that IQR is Q3-Q1. From a statistical point of view, outliers are those points beyond whiskers.

**Figure 3.**
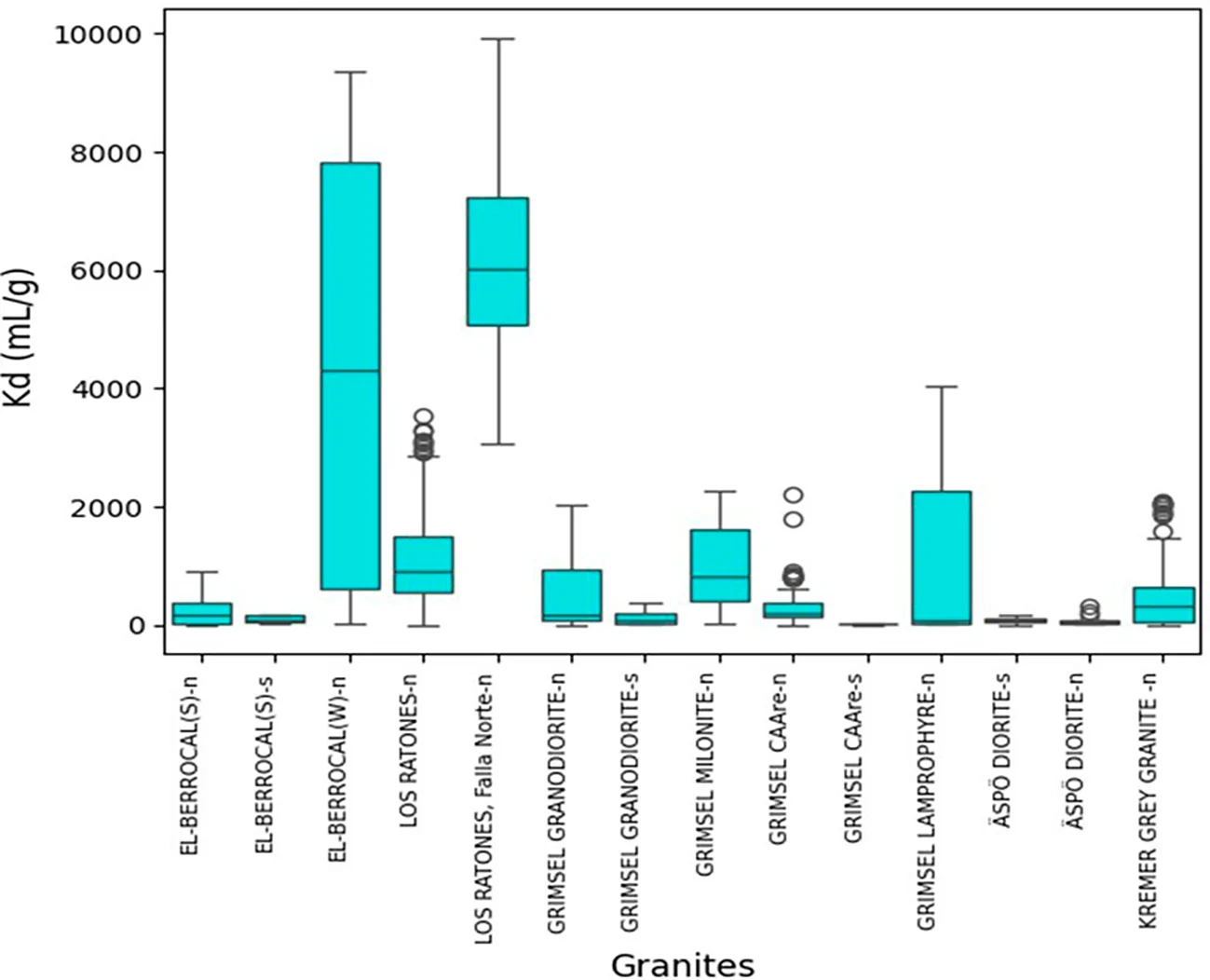
Boxplot of K _d_ by type of granite samples. Complete dataset for crystalline rocks. “n” or “s” refer to non-saline or saline water, as explained in the text.

Several materials have many high outlier points, which could indicate casual extreme adsorption values from a statistical perspective. These must be analyzed and not simply overlooked. Because Cs sorption is not linear, very high K _d_ values can be observed at the lowest Cs concentrations, which are indeed real and cannot be discarded.

Figure 3 shows the data divided according to the site and the type of material selected. Apart from fresh rock, we also weathered the rocks and fracture-filling material. In Grimsel, we distinguish between fresh rock, mylonite, and lamprophyre. Without detailed information on the different materials, mylonite is a type of rock produced when pre-existing rock is intensely deformed and ground down along fault zones, leaving it very fine-grained and fractured, with a laminar appearance. Lamprophyres are dark, fine-grained igneous rocks that usually occur as dikes, sills, or other small intrusions. They are generally rich in mafic minerals, such as biotite, amphibole, and pyroxene, with feldspar mostly appearing in the groundmass rather than as large crystals.

They were considered of interest because they were present in the experimental tunnels of Migration and FEBEX in the fractured zones. The sample of the altered granite of El-Berrocal (Berrocal (W)) corresponds to a hydrothermally altered rock near a mineralized quartz vein. This has been subject to sericitization of feldspars (albite), chloritization of biotite, and carbonate precipitation. This sample contained a large number of phyllosilicates.

The suffix -n/−s after the name of the sample in the x-axis of the boxplot indicates whether the water used in the experiments was saline (s) or not (n). The threshold of the ionic strength considered was 0.01 M, (being saline ≥ 0.01 M).

As shown in the boxplot, the distribution coefficients vary widely across sites and rock types. The materials with the highest K _d_ values are the altered El-Berrocal granite and the Los Ratones northern faults (Falla Norte) material from the SR-3 borehole. These samples are not fully representative of fresh granite but can also affect the overall retention properties of the rock. If more information on fresh granite is required, these samples can be omitted from detailed analysis.

El-Berrocal(W) presented the highest spread and a median K _d_ of approximately 4500 mL·g ^−1^. The *Los Ratones* northern fault material of the Los Ratones shows a median K _d_ of approximately 6000 mL·g ^−1^, with values also widely spread. The rest of the samples presented intermediate K _d_ values (< 2000 mL·g ^−1^) with some higher outliers. The material showing less retention is related to Äspo diorite. The granites from the Grimsel Central Aare and the reference El-Berrocal also have low median values.

In general, it can be stated that under certain experimental conditions, rocks such as lamprophyre and mylonite have more retention capability for Cs but show more variability, which can be explained by the type and number of experiments performed, whereas others such as diorite and central Aare granite have weaker adsorption.

*4.1.2.3 Correlation matrix*

With all available data, the calculation of correlation matrices can be performed according to the three main blocks (experimental, water properties, and solid properties), and different methodologies such as the Pearson or Spearman approximation can be used. This kind of approximation helps identify which variables are strongly related to not using duplicates in a model.

Pearson correlations are based on the linear relationship between two variables, assuming that both variables are normally distributed and have a linear relationship, which is not always the case in this study. It computes the correlation based on the covariance and standard deviations. The values ranged between −1 and 1. If the value is −1, the relationship is perfectly linear with a negative slope; if it is 1, it is perfectly linear.

On the other hand, Spearman’s correlations are rank-based correlations that measure the monotonic relationship (not necessarily linear) between two variables. No assumptions were made regarding normality or linearity. It converts the data into ranks and then applies Pearson correlation to the ranked values. Again, the data range from −1 to 1, expressing decreasing/increasing monotonic relationships.

These types of matrices can also be expressed as heatmaps, where the visual information immediately makes it easy to quickly identify relationships between variables. Values are shown as colors where darker/brighter colors show stronger correlations and neutral/lighter colors show weak or no correlation.

An example of the calculated Pearson correlation matrix for all granite data is shown in Figure 4. It must be pointed out that the correlation of certain parameters may improve when subsets are selected (i.e., eliminating fracture filling materials, selecting a range of Cs concentrations, etc.). However, for all experimental conditions, the highest (negative) correlation for K _d_ exists with the initial Cs concentration, meaning that sorption clearly decreases as [Cs] increases, and sorption is non-linear. A very small negative correlation was found between the solid-to-liquid ratio and grain size.

**Figure 4.**
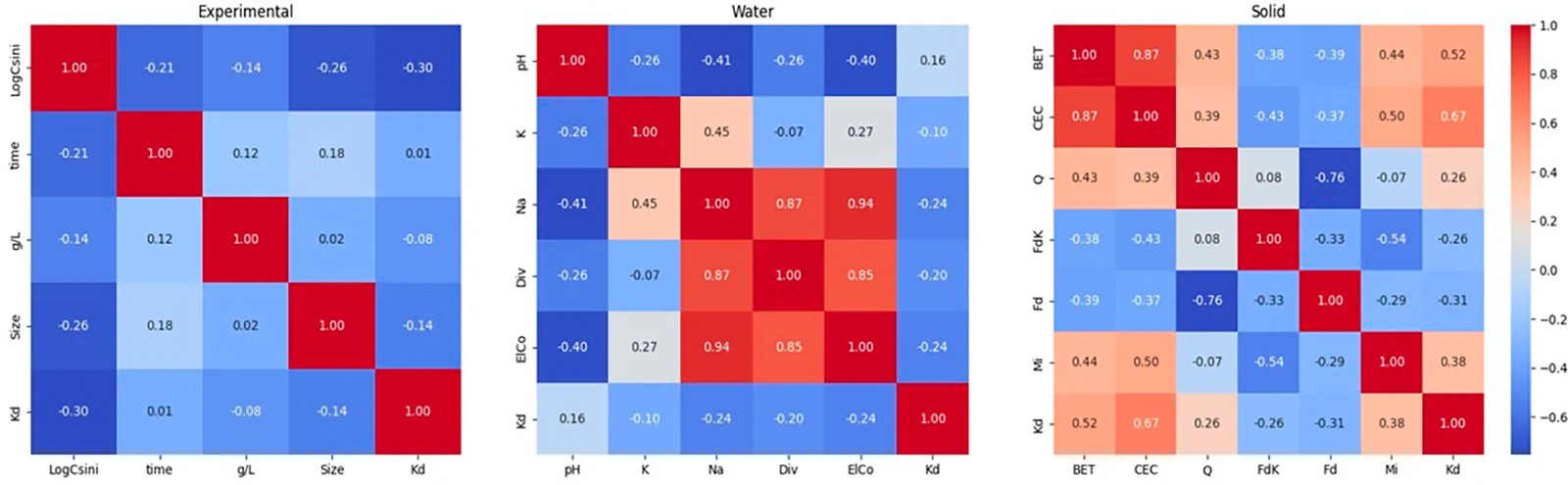
Example of the correlation between experimental parameters. “Div” refers to the sum of Ca and Mg ions, ElCo to the electrical conductivity of the water.

The correlation matrix for the water chemistry properties shows a negative correlation between K _d_ and the concentrations of the mayor ions K, Na, Ca, and Mg (and therefore with the electrical conductivity), indicating that sorption decreases as the ionic strength of the solution increases. The effect of ionic strength is expected, because the main mechanism of cesium sorption is ionic exchange. Nevertheless, a positive correlation was observed with the pH; however, it must be highlighted that most of the data were collected under neutral to alkaline conditions, and more data would be needed under acidic conditions.

An important correlation of K _d_ with the BET and CEC of the materials was observed, which is also expected to be positively correlated with the content of micas and quartz, but negatively correlated with feldspars. The (unexpected) positive correlation with quartz can be explained by considering that the two most degraded samples (El Berrocal (W)) and Los Ratones northern faults, which present the highest sorption, contain very high quantities of quartz apart from some clays. Analyses indicate that the initial concentration is the experimental parameter that most affect (negatively) K _d_ values; in general, the ion concentration in the aqueous phase also negatively affects the K _d_ values, and the important parameters that positively affect K _d_ are CEC, BET, and the presence of micas.

### 4.2 Cs sorption data in minerals

***4.2.1 Data visualization***

Figure 5 shows all data for Cs sorption in the minerals. Again, Cs concentration plays a critical role in its sorption. Quartz is a mineral with less sorption, whereas micas (biotite and muscovite) have high sorption, but are also comparable with that of potassic feldspar (FdK), for which we observed a negative correlation with Kd in the K _d_. This is an interesting point; however, experimental data must be checked, and strong conclusions cannot be drawn, considering that only one material and a series of data are currently available for FdK in the database.

**Figure 5.**
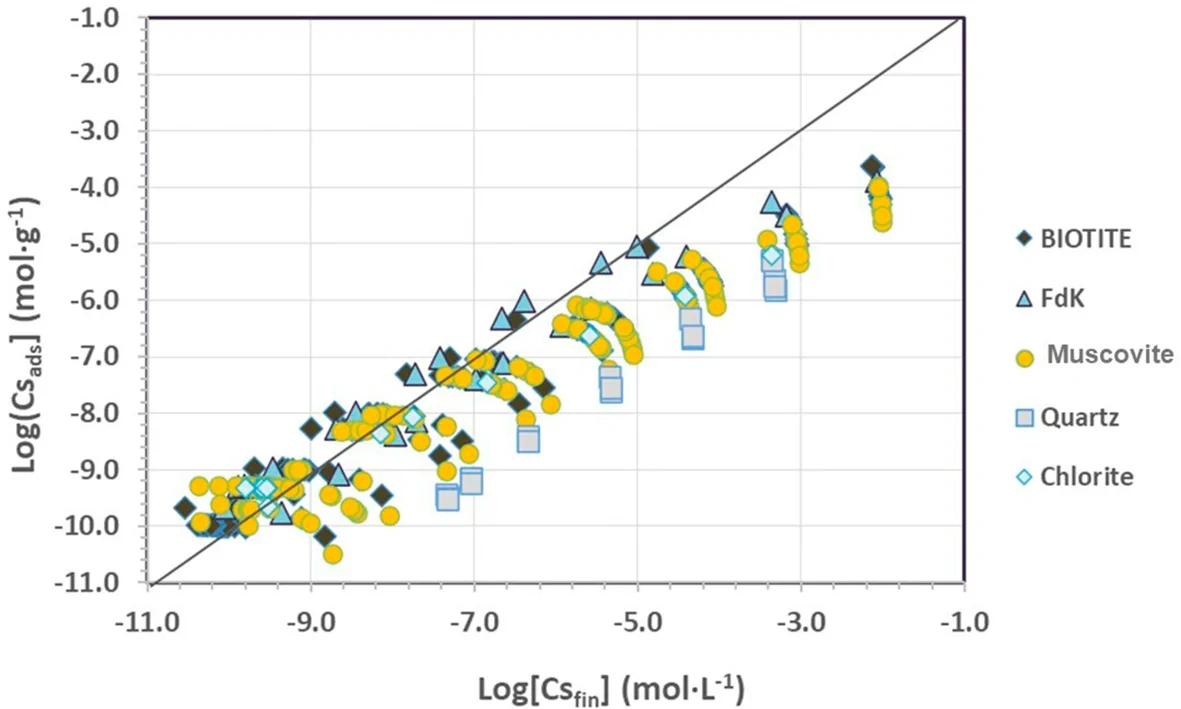
Log-Log representation of Cs sorption data in granite minerals.

***4.2.2 Basic statistics***

*4.2.2.1 Descriptive statistics*

Table 3 presents the descriptive statistics of the experimental database of Cs sorption in minerals. For the mineral sorption data, Table 3 shows the wideness of the explored experimental conditions.

**Table 3.** Descriptive statistic of the mineral data.

index	time (days)	[Cs]	pH	[K] (mg/L)	[Na] (mg/L)	[Ca + Mg] (mg/L)	BET (m ^2^/g)	CEC (meq/100 g)	Size (mm)	g/L	K _d_ (mL/g)
**mean**	12.72	4.96E-04	6.89	265.53	256.51	113.46	3.51	0.78	0.21	9.98	784.64
**std**	7.48	2.00E-03	0.61	932.50	633.70	306.25	2.35	0.47	0.21	0.24	1212.97
**min**	0.17	7.37E-10	5.33	0.00	0.00	0.00	0.40	0.05	0.06	9.70	2.55
**0.25**	7.00	5.41E-09	6.56	0.29	8.80	6.50	0.83	0.58	0.10	9.86	29.66
**0.5**	14.00	5.00E-07	6.85	5.40	9.20	8.00	3.98	0.97	0.10	9.91	176.44
**0.75**	14.00	4.95E-05	7.03	13.00	9.90	10.33	5.55	1.07	0.50	10.00	1344.86
**max**	93.00	9.85E-03	9.47	3910.00	2299.00	1000.00	8.00	1.70	1.00	11.70	12073.42

Experiments were carried out from hours to 93 days, with a variable Cs concentration of approximately 8·10 ^−10^ to 1·10 ^−2^ M approximately. The grain size (< 1 mm) and solid-to-liquid ratio (~10 g·L ^−1^) ranges were more limited than in the case of crystalline rock data.

*4.2.2.2 Box plots*

Figure 6 shows boxplots of the K _d_ values for the different minerals. The distribution coefficients extend up to ~12000 mL·g ^−1^, even if all values above mL·g ^−1^ are outliers. Biotite-n shows a wide range of values with a median near 1000 mL·g ^−1^ and many outliers exceeding 7000 mL·g ^−1^. Under saline conditions, Biotite-s showed much smaller K _d_ values than Biotite-n, and some outliers were present. Chlorite-n has a large spread, with a median of approximately 1000 mL·g ^−1^ and possible outliers. FdK-n presented a higher median (approximately 2000 mL·g ^−1^) than the others, with a certain spread, whereas FdK-s presented a much smaller and tight distribution.

**Figure 6.**
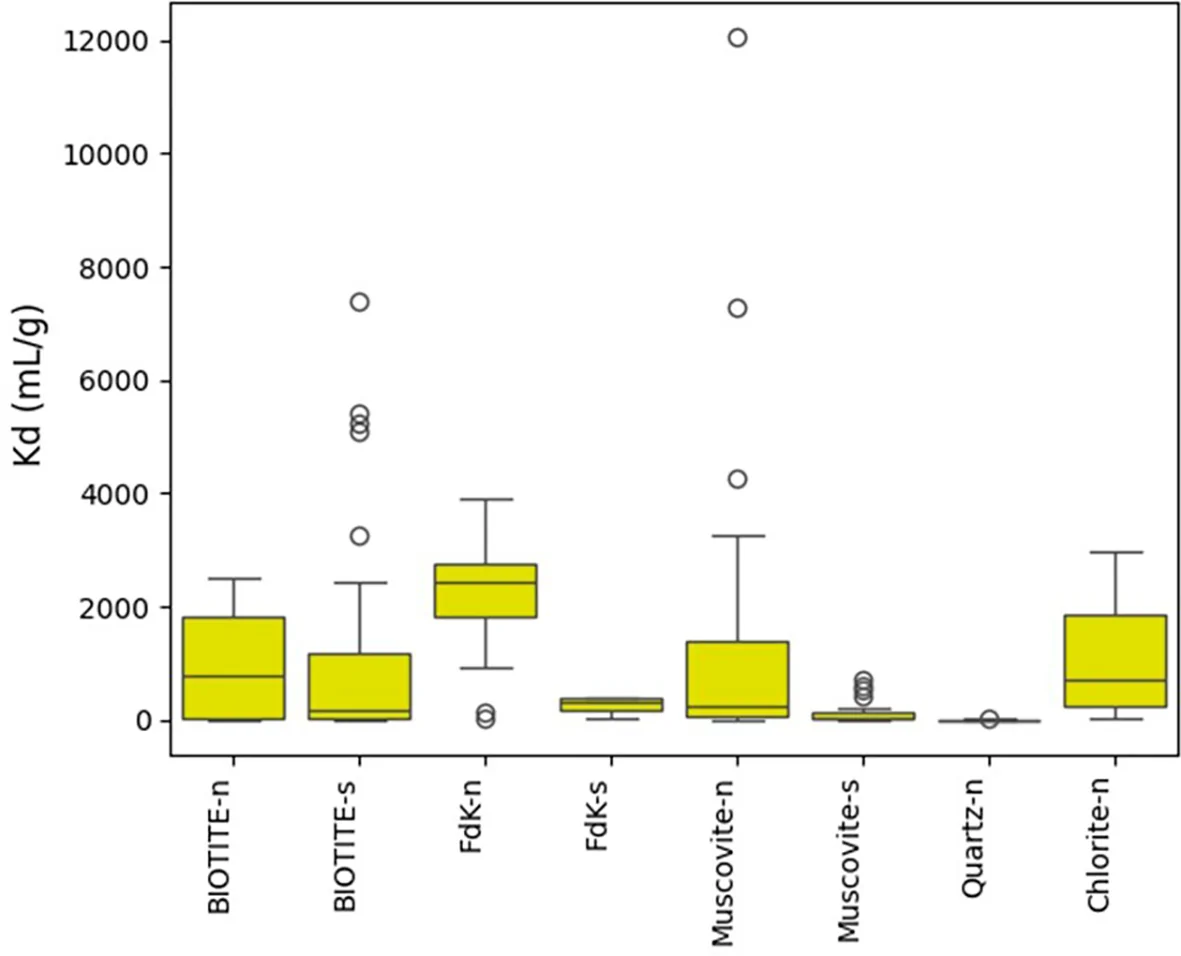
Boxplot of K _d_ by type of mineral. “n” or “s” refer to non-saline or saline water, as explained in the text.

Muscovite-n has a wide distribution, with many outliers, some reaching above 12000 mL·g ^−1^. In contrast, Muscovite-s showed very small values and a tight distribution. Finally, quartz-n showed a very low K _d_ with few outliers.

Again, all the distribution coefficients obtained in non-saline waters (−n) tend to show higher K _d_ values with more variability, whereas tests performed under saline conditions (−s) have lower and tighter distributions. This confirms the role of salinity in Cs retention, as observed in studies on rocks. Quartz shows the lowest K _d_ among all, and muscovite and biotite are the samples exhibiting extreme upper outliers. All the samples show K _d_ within a similar range, excluding FdK, as mentioned above, and quartz with the lowest values. The larger source of uncertainty still comes from the non-linear sorption of cesium in most of these materials.

***4.2.3 Sorption modelling***

The previous paragraphs show the possible use of the provided database, focusing on purely descriptive aspects; however, the use of detailed experimental databases may facilitate the implementation of sorption modelling. The objective of this work is not to provide an in-depth modelling study but rather to offer indications of how the data can be used.

The results of the statistical analysis can be used to define representative parameter ranges and distributions, identify data subsets suitable for modelling, evaluate the potential predictive capacity of existing sorption models against empirical trends, and support uncertainty propagation studies in future modelling tasks. Furthermore, the structure of the database is useful for the selection of representative datasets based on material type, experimental conditions, and other criteria.

Mechanistic models explicitly consider interactions at the mineral-water interface. The two most commonly adopted approaches are surface complexation models (SCMs) and ion exchange models (IEMs), both of which are equilibrium-based. SCMs describe the complexation of ions on variable-charge surfaces and explicitly account for electrostatic effects through formulations such as the constant-capacitance model (CCM), diffuse-layer model (DLM), and triple-layer model (TLM). In contrast, IEMs exhibit reversible ion exchange on fixed-charge sites, with selectivity governed primarily by electrostatic interactions.

In this study, the IEM is particularly interesting because cation exchange is the main Cs retention mechanism, whereas surface complexation is a minor retention mechanism and will not be described here.

Cs sorption is nonlinear; therefore, we can assume the existence of more than one adsorption site in granite or other minerals. This behavior is typical for Cs sorption in illite (and in general micaceous materials), and has been modelled with three types of sorption sites ( Poinssot et al., 1999; Bradbury and Baeyens 2000). Modelling is mostly carried out with minerals that have been exchanged in Na, a practice that has become the standard methodology. However, this approach introduces uncertainty because the selectivity coefficients are experimentally determined only for Na, whereas those for other cations are calculated, hindering the accurate evaluation of competitive sorption effects.

In Na-exchanged materials, Na ^+^ is the (unique) cation balancing the surface negative charge, which can be substituted by another cation present in the solution (cation B, with valence zB z = 1 in the case of Cs ^+^). Considering that Na ≡ X represents the exchange site at the Na mineral surface, the exchange reaction is expressed as:

$$\left(\mathrm{Na}\equiv X\right)+Cs^{+}↔\left(\mathrm{Cs}\equiv X\right)+Na^{+}$$

If the main exchanger would be Ca, the reaction is:

$$\left(\mathrm{Ca}\equiv X_{2}\right)+2Cs^{+}↔2\left(\mathrm{Cs}\equiv X\right)+Ca^{2+}$$

Very few studies have reported the exchange complex of granite material, and in general, the main exchangeable cation in granitic materials seems to be Ca. In the present database, some data correspond to exchanged rocks or minerals with Na or K, but not with Ca, which is an important observation raised from the analysis of the database.

An illustrative example of the possible application of the database for modelling purposes is explained below. In the database, information can be extracted from seven different biotites: two commercials and one granite at the *El-Berrocal* and *Los Ratones* sites. A biotite from *El-Berrocal* (denominated Biotite 3F, Bio3F) was selected as an example because it was used in several tests, including sorption isotherms and sorption edges, and was also tested after its exchange with Na and K.

The experiments on raw Bio3F were carried out in both low- and high-salinity waters, while experiments with the exchanged material were performed in the corresponding electrolytes (NaClO _4_ or KCl) at two ionic strengths (0.1 and 0.01 M).

Figure 7 presents the sorption isotherms for both the raw and homoionized samples. To facilitate the description of the behavior of K _d_, data were expressed as the logarithm of the distribution coefficient (log (K _d_)) *vs* the logarithm of the cesium remaining in solution (Cs _fin_).

**Figure 7.**
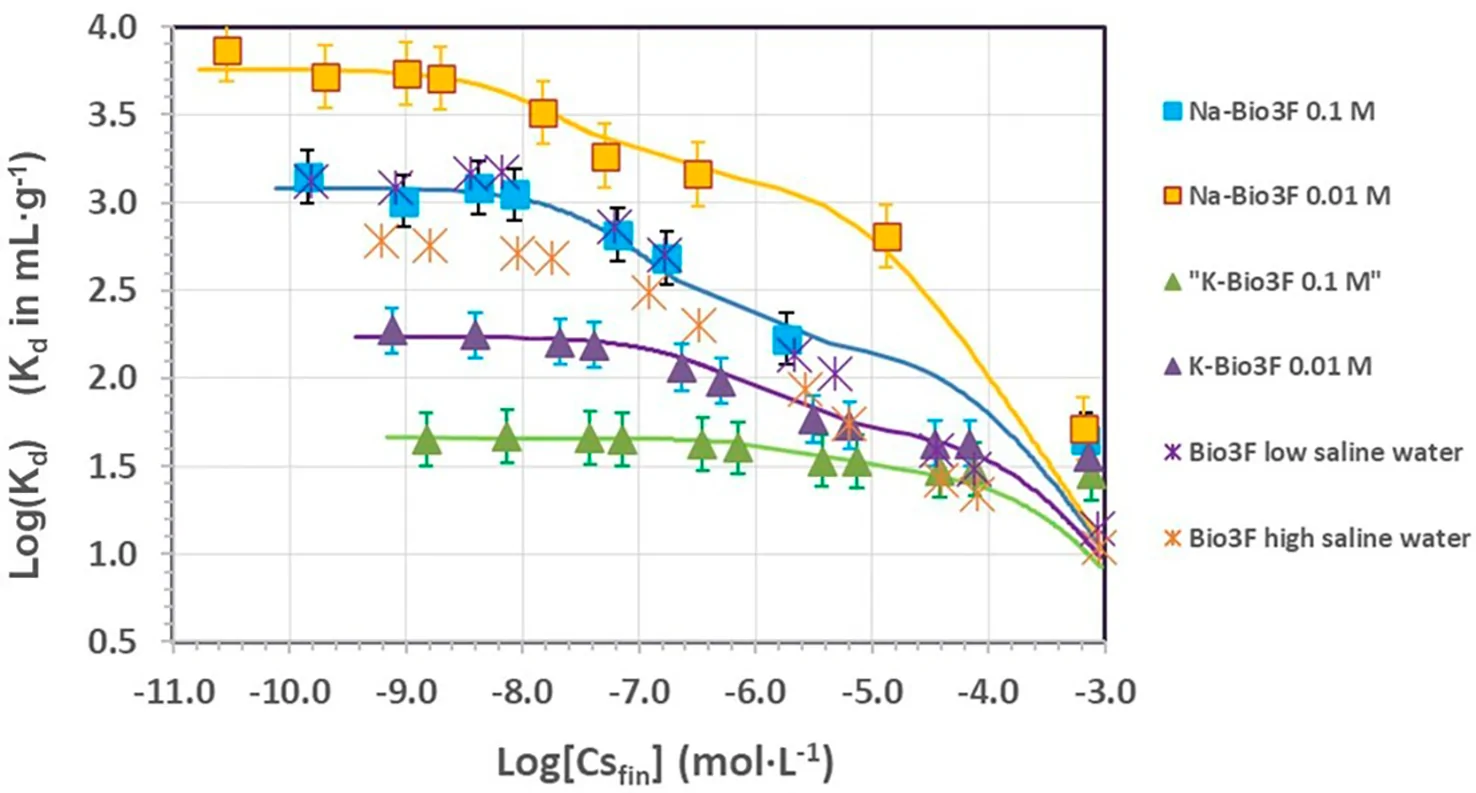
Sorption isotherms of El-Berrocal biotite (3F) raw and exchanged in Na and K. Squares corresponds to Na-exchanged biotite at (■) 0.1 M and (■) 0.01 M in NaClO _4_; triangles corresponds to K-exchanged biotite at (▲) 0.1 M and (▲) 0.01 M in KCl; crosses correspond to the untreated material in ( *) low and ( *) high saline waters.

Results show that Bio3F exhibits maximum cesium adsorption when homoionized in Na in NaClO _4_ at 0.01 M. However, cesium uptake by raw biotite in non-saline water is comparable to that of Na-biotite at an ionic strength of 0.1 M, suggesting that sodium does not strongly compete with cesium for sorption sites.

In contrast, cesium sorption onto raw biotite decreased under natural saline conditions. Potassium exerts a stronger effect; the presence of K reduces cesium adsorption by more than an order of magnitude, with higher potassium concentrations leading to progressively lower sorption. As previously discussed, the nonlinear behavior of cesium sorption in micas can be interpreted by assuming the presence of three distinct sorption sites. The first site was the most selective for cesium and accounted for the highest sorption values, although its density was relatively low. Based on the isotherm shape, according to the first saturation of sorption sites, a density of approximately 0.01 μeq/m ^2^ was estimated. Interestingly, this value appears to be consistent across most of the samples analyzed; therefore, it was considered a fixed parameter in the modelling. The remaining sorption capacity, in terms of site density, was estimated using the experimentally measured CEC and N _2_-BET surface area. The densities of the second and third sites were more difficult to determine, but after detailed analysis of all biotite data, we found that assigning 10% and 90% of the total sorption capacity to the second and third sites, respectively, provided the best fit to the experimental data. It must be mentioned that, probably because of the quite low CEC of these materials, sorption isotherms could also be well simulated using only two sites, making the model easier with a similar performance.

Figure 7 shows that a satisfactory fit was obtained in all the cases. In the case of Na-biotite, for the first site, we obtained a logarithm of the selectivity coefficient between Cs and Na ( Cs of 6.1 ± 0.3; and for the second and third sites, it was 3.4 ± 0.2 and 3.1 ± 0.1, respectively. In contrast, K-biotite exhibits lower adsorption, reflected by the lower selectivity coefficients between Cs and K ( Cs . For the first site, the logarithm of the selectivity coefficient is almost two orders of magnitude lower than that for Na-biotite (4.4 ± 0.1), while the second and third sites yield values of 2.3 ± 0.3 and 1.9 ± 0.3, respectively. The larger uncertainties observed, particularly at higher ionic strengths, arise from the flatness of the sorption isotherm, which again indicates that a two-site model can also be adopted.

## 5. Discussion

A proof-of-concept for a methodology for building interoperable sorption databases is presented to demonstrate the value of comprehensive, structured, and flexible data for analyzing radionuclide sorption data. Although the current database includes a relatively large dataset (~1000 records), it still contains gaps, such as limited data under extreme pH conditions and missing information on certain minerals and exchanged forms (especially Ca-exchanged). However, this database is openly available and intended as a continuously evolving resource, encouraging contributions and the early integration of structured data practices in experimental research.

One issue associated with experimental databases is the application of advanced predictive tools. For the sorption data, we aimed to predict sorption under different environmental conditions. The applications of artificial intelligence (AI) and machine learning (ML) have become increasingly popular in recent years, including in the field of radioactive waste management.

ML models generally require thousands to tens of thousands of examples to achieve robust performance and to build generalized models. Importantly, ML approaches remain largely empirical: they can highlight correlations, but do not inherently provide mechanistic or causal insights. AI models are typically strong at interpolation within the training domain, but less reliable when extrapolating to new conditions, such as untested mineral types, concentration ranges, or additional effects (e.g., colloids or organics). Consequently, human expertise remains essential for interpreting AI results.

In contrast, mechanistic models are grounded in physicochemical processes and are especially useful for interpreting results, particularly when the data are curated and complete. Given the size of the present database, the most effective strategy is to combine domain expertise in physical and geochemistry. Nevertheless, parameters derived from geochemical modelling can be used to constrain ML algorithms with mechanistic equations, thereby enhancing both predictive power and interpretability, advancing the hybrid approach.

## 6. Conclusions

This work introduces a structured and interoperable sorption database framework that not only compiles experimental data but also links sorption behavior (K _d_ values) to key physicochemical parameters, enabling a deeper analysis of controlling factors.

This study underscores the importance of standardized, well-documented data sharing from the perspective of data producers, particularly in collaborative research contexts. The database supports long-term data preservation, reuse, and scientific collaboration by adhering to FAIR principles and is openly accessible via Zenodo. Its interoperable design allows total integration with other databases and flexible reuse without additional tools. Ultimately, this proof-of-concept highlights the value of embedding data management frameworks at the start of experimental work and sets the foundation for iterative knowledge development and improved predictive capabilities in radionuclide behavior studies.

## Ethics and consent

Ethical approval and consent were not required.

## Data Availability

The experimental dataset is available in ZENODO under an Open Licence: CC-BY-4.0: Missana and García-Gutiérrez M. (2025). Proof of Concept for Sorption Data Management: The Case of Cs Sorption in Crystalline Rocks (DATA SET Version v1.0). https://zenodo.org/records/17930595. (doi: 10.5281/zenodo.17930594).
