# Supplementary material for: A methodological framework for building interoperable sorption databases: application to cesium sorption in crystalline rocks
Source: Open Res Eur. 2026 May 26;6:175. [Version 1] doi: 10.12688/openreseurope.23714.1 (PMC13681308; doi:10.12688/openreseurope.23714.1)
Supplement: Peer review history

# Reviewer report: Reviewer response for version 1

**DOI:** 10.21956/openreseurope.25673.r78205

**Published:** 2026-08-20

**Authors:** Crina Bucur

**Affiliations:** 1 Institute for Nuclear Research Pitesti, Pitesti, Romania

While current radionuclide sorption databases offer valuable domain-specific data and modeling capabilities, they remain fragmented and vary in completeness, highlighting an urgent need for standardized, quality-assured data curation and structured experimental workflows to ensure consistency, traceability, and reusability. This work proposes a structured and flexible methodological framework for constructing experimental radionuclide sorption databases that link Kd values with detailed experimental, hydrochemical, and mineralogical parameters to enable versatile statistical analysis and sorption modeling.

 The proposed framework is demonstrated for cesium sorption in crystalline rocks. Cesium was selected due to its simple chemistry and well-documented sorption behavior, including experimental work performed at CIEMAT over the last few decades. Moreover, selecting crystalline rocks as a test case provides a wide range of chemical environments (driven by their mineralogical diversity and heterogeneity), which significantly enhances data analysis.

 The two sets of experimental data (on individual minerals and granitic rock, respectively) included in the database available in ZENODO, contain over 1,000 records of cesium Kd along with the relevant parameters describing experimental conditions under which these values were obtained.

 For both sets of data, a preliminary exploratory study using descriptive statistics, boxplots, and correlation matrices was performed to demonstrate data usability, assess Kd variability and parameter correlations, and direct future modeling of cesium retention mechanisms.

 The study introduces an open, FAIR-compliant, and evolving sorption database framework that links Kd values to key physicochemical parameters, fostering long-term data reuse and laying the foundation for hybrid predictive approaches that combine machine learning with expert mechanistic geochemical modeling.

**General comment**: 

 Overall, the manuscript is written in clear and high-quality scientific English appropriate for the geochemical field. However, regarding the authors' note that the Kd errors were not added in the first version of the database, but will be included “asap”, it is reccommended to replace the colloquial term "asap" with a formal formulation, such as: “will be incorporated in subsequent versions” or “planned for future updates”

Is the rationale for developing the new method (or application) clearly explained?

Yes

Is the description of the method technically sound?

Yes

Are the conclusions about the method and its performance adequately supported by the findings presented in the article?

Yes

If any results are presented, are all the source data underlying the results available to ensure full reproducibility?

Yes

Are sufficient details provided to allow replication of the method development and its use by others?

Yes

Reviewer Expertise:

Radioactive waste disposal, radionuclide migration

I confirm that I have read this submission and believe that I have an appropriate level of expertise to confirm that it is of an acceptable scientific standard.

# Reviewer report: Reviewer response for version 1

**DOI:** 10.21956/openreseurope.25673.r78203

**Published:** 2026-08-20

**Authors:** Norbert MAES

**Affiliations:** 1 SCK CEN - Belgian Nuclear Research Centre, Boeretang, Belgium

This paper proposes a simple to use framework for building and sharing sorption databases. This is particularly relevant for the Safety assessment of nuclear waste repositories. A proper safety assessement should be based on carefully selected high quality data. Such data are typically gathered in databases. 

 The methodology presented here couples the Kd values with the main physicochemical parameters which makes the database also useful for geochemical analysis purposes and tailoring of data selection towards specific conditions. The methodology presented is easy to apply and does not require specific computer tools (straigthforward Excel table is used here)  which can be seen as a pro but also as a con. However, the use as Excel as database format is not the core idea of the methodology, the core idea is to link sorption values to the details on i) experimental conditions, iii) water chemistry and iii) solid characteristics and this should be independent of the tool used to build the database. 

 Nevertheless, there are few shortcomings to the current excel version of the database. 

 - as the database relates to the FAIR principle, we miss reference to the primary data source of the entries. hence there is no real traceability. 

 - the tabs for granite and minerals are not similar. In the granite tab, part of the water chemistry data are missing (no data for anions....)

 -  The current data entries are targeted towards Cs, with chemistry details targeted also towards the "chemical behaviour of Cs". If a database for U is constructed, other water chemistry details would be needed as well... The point i want to make is that it would be better to create an data entry sheet that is applicable for all types of RN, minerals. If all data sheets have the same format, it is much easier for data extraction towards other applications. 

 - I also miss a column with auxiliary information. Sometimes there are certain details in the primary data source that are worthwhile to mention. 

 - for the time being details on experimental uncertainty are not taken up (but will be done in future as mentioned by the authors). That is indeed an important aspect. 

 Some details to change in the text: 

 "The Belgian agency for radioactive waste, ONDRAF/NIRAS," --> The Belgian agency for radioactive waste and enriched fissile materials... 

 "Crystalline rocks (CR) are suitable for radioactive waste storage, and the first repositories in Europe, Finland, and Sweden **were** built in CR. " --> are

 "The sheet *Minerals* the sheet *Granite* contained 436 and 577 data lines" --> the sheet Minerals and the sheet Granite contain.... (don't use past tense)

 "C _in_ is the initial contaminant concentration and C _fin_ is the quantity of contaminant remaining in the liquid. V is the volume of water and m is the mass of the solid. The quantity C _in_-C _fin_ represents the contaminant adsorbed on the solid." --> add units for C, V, m... 

 "The considered limit is 0.01 M (n < 0.01 M; s ≥ 0.01 M)." --> consistent notation of concentration: M or mol.L-1

 " **FIN [Cs]:** final concentration of cesium in solution in mol·L ^−1^ **(**Log (Cs _fin_) was calculated)." --> also the FIN[Cs] is calculted in the database. it uses the Kd value and the V/M to calculate the FIN[Cs].  from an experimantalist view, I would report the real measured values: V/M, FIN[Cs], INI[Cs] as Cads and Kd are calculated from them. Here in the database it is the opposite... But indeed, for building a database from literature data, sometimes you only have INI[Cs], V/M and Kd but not the FIN[Cs]... 

 "The Äspö Hard Rock Laboratory (Äspö HRL) **was** a research facility" --> is. (try to avoid use of past tense...)

 "When available, the composition of the solution upon equilibrium with solids was determined ( Missana et al., 2019)" --> in the current form of the database, the reader can not know if the mentioned solution details are linked to the solution applied before interaction or at the end. This is indeed important information. Therefore I proposed in my comments to the current database form to add an extra column where this kind of information can be mentioned. 

 "Figure 1" --> I prefer a plot logCsads vs LogCsFin (authors also mention this in the paragraph preceding the figure that it is also the most prefered way of presenting). 

 "In Grimsel, we distinguish between fresh rock, mylonite, and lamprophyre. Without detailed information on the different materials, mylonite is a type of rock produced when pre-existing rock is intensely deformed and ground down along fault zones, leaving it very fine-grained and fractured, with a laminar appearance. Lamprophyres are dark, fine-grained igneous rocks that usually occur as dikes, sills, or other small intrusions. They are generally rich in mafic minerals, such as biotite, amphibole, and pyroxene, with feldspar mostly appearing in the groundmass rather than as large crystals.

 They were considered of interest because they were present in the experimental tunnels of Migration and FEBEX in the fractured zones. The sample of the altered granite of El-Berrocal (Berrocal (W)) corresponds to a hydrothermally altered rock near a mineralized quartz vein. This has been subject to sericitization of feldspars (albite), chloritization of biotite, and carbonate precipitation. This sample contained a large number of phyllosilicates." --> I advice to move this section to paragraph 3.2.2 where you discuss the selected materials. The details provided fit better in that section. 

 "Table 2 and 3" --> add units to the column header [Cs] (furthermore is it Cs INI of FIN - i think INI), add explanation to the values 0.25/0.5/0.75 in the first column. 

 "Figure 4" mention that this is pearson correlation

 "but are also comparable with that of potassic feldspar (FdK), for which we observed a negative correlation **with Kd in the K _d_.**" --> confusing... furthermore, better explain that you relate this to the FdK portion present in the granitic samples. 

 "An illustrative example of the possible application of the database for modelling purposes is explained below. " --> Please explain in a few sentences the exact objective that you wanted to achieve with this modelling. Now you explain a lot on the how, but not on the why... So, selected data sets are used in a modelling excercise to extract Ksel for 3 different sorption sites (and their relative distribution), is this correct? It deserves attention to clearly describe your objective as to show the power of your methodology... 

 In the discussion there are 2 paragraphs on ML. Although I agree with the content, they do not immediately discuss the content of the presented work. I'm ok to mention it but I would rather see a deeper elaboration on the how you see the further evolution of the database, possible improvements etc.

Is the rationale for developing the new method (or application) clearly explained?

Yes

Is the description of the method technically sound?

Yes

Are the conclusions about the method and its performance adequately supported by the findings presented in the article?

Yes

If any results are presented, are all the source data underlying the results available to ensure full reproducibility?

Yes

Are sufficient details provided to allow replication of the method development and its use by others?

Yes

Reviewer Expertise:

Geochemistry, specifically the behaviour of radionuclides in barries - sorption, diffusion through clays/cements

I confirm that I have read this submission and believe that I have an appropriate level of expertise to confirm that it is of an acceptable scientific standard.
